# Optimal control strategies on COVID-19 infection to bolster the efficacy of vaccination in India

**DOI:** 10.1038/s41598-021-99088-0

**Published:** 2021-10-11

**Authors:** Ashutosh Rajput, Mohammad Sajid, Tanvi, Chandra Shekhar, Rajiv Aggarwal

**Affiliations:** 1 Department of Mathematics, Deshbandhu College, University of Delhi, New Delhi, 110019 India; 2 Department of Mechanical Engineering, College of Engineering, Qassim University, Buraidah, Al Qassim Saudi Arabia; 3 DBC i4 Centre, Deshbandhu College, University of Delhi, New Delhi, 110019 India

**Keywords:** Diseases, Mathematics and computing

## Abstract

The Novel Coronavirus which emerged in India on January/30/2020 has become a catastrophe to the country on the basis of health and economy. Due to rapid variations in the transmission of COVID-19, an accurate prediction to determine the long term effects is infeasible. This paper has introduced a nonlinear mathematical model to interpret the transmission dynamics of COVID-19 infection along with providing vaccination in the precedence. To minimize the level of infection and treatment burden, the optimal control strategies are carried out by using the Pontryagin’s Maximum Principle. The data validation has been done by correlating the estimated number of infectives with the real data of India for the month of March/2021. Corresponding to the model, the basic reproduction number ${\mathcal {R}}_0$ is introduced to understand the transmission dynamics of COVID-19. To justify the significance of parameters we determined the sensitivity analysis of ${\mathcal {R}}_0$ using the parameters value. In the numerical simulations, we concluded that reducing ${\mathcal {R}}_0$ below unity is not sufficient enough to eradicate the COVID-19 disease and thus, it is required to increase the vaccination rate and its efficacy by motivating individuals to take precautionary measures.

## Introduction

COVID-19 is a contagious respiratory infection caused due to a new coronavirus called SARS-Cov-2. The effect of COVID-19 infection may vary from person to person. The illness from mild to moderate can be observed in most of the infected individuals and they may recover themselves without getting hospitalized. COVID-19 infection can be transmitted through the infected individuals via direct or indirect close contacts. Transmission occurs majorly through asymptomatic infectives. Individuals with other comorbidities or with a compromised immune system are more prone to develop illness due to COVID-19 disease.

By the end of March/2021, COVID-19 had hazardously affected around 106 different countries and territories and hence, caused over 133 million confirmed cases. Out of which, more than 2.9 million infected people have died due to COVID-19 infection. According to this, we may say that more than 21 infected individuals are dying out of 1000 individuals infected with COVID-19 disease. As reported by the World Health Organization, community transmission was initially the cause for the transmission of COVID-19 infection in India, but later the cluster of cases has become a major concern. The Ministry of Health and Family Welfare has discontinued various activities at some major public places and social gatherings to control the transmission of COVID-19 infection. In the beginning of April/2021, the total confirmed cases of COVID-19 disease had already crossed 12 millions. Also, the total disease induced deaths have crossed 164,108. According to WHO, more than 70% deaths of COVID-19 infectives occurred due to the infection with other comorbidities.

According to the World Health Organization^34^, some of the common symptoms of COVID-19 disease are high fever, dry cough, sneezing, breathlessness and pneumonia. To control the transmission of COVID-19 infection among individuals, many countries have initially implemented complete or partial lockdowns in the year 2020. However, it was not that effective due to rapid increment in the number of hospitalized infectives and thus, increased the number of COVID-19 cases. In total COVID-19 has affected over 215 countries all over the world. However, the major devastation has been experienced by approximately 106 countries. Scientists from all over the world are collaborating to bring us various treatment procedures and vaccination to reduce the burden of COVID-19 pandemic. Nowadays vaccination has become a crucial player for the battle against COVID-19 infection. The health care authorities are highly encouraging the individuals to receive the vaccination in the precedence order. However, taking vaccination does not ensure the complete protection of an individual but reduces the risk of infection from COVID-19 disease. Thus, even after taking vaccination it is essential to implement the non-pharmaceutical inventions such as infection prevention (wearing masks and sanitizing hands), quarantine, social distancing, contact tracing and isolation of infectives. According to the World Health Organization, prevention measures such as mask wearing and keeping one meter distance must be a part of daily routine for each individual to avoid the transmission of COVID-19 infection through infectives. However, the appropriate use and cleaning techniques are also necessary to maintain the masks as effectively as possible.

It has become an important concern to predict and analyze the severity of COVID-19 for both health care authorities and researchers. Mathematical modeling provides an efficient way to analyze the real world problems such as the prediction of COVID-19 prevalence under certain assumptions. In order to model the real life problems more practically, a number of mathematical models have been proposed by various researchers with the time dependent parameters such as time dependent treatment rates and transmission rates to study the transmission dynamics of infectious diseases^1,25–28^. Many researchers are working in this direction to predict the severity and analyze the transmission dynamics of COVID-19^2,5,10,12–14,16,17,21,22,29–31,33,37^. Pang et al.^18^ have proposed a mathematical model for COVID-19 by considering transmission in three different stages on the basis of different control measures. They have concluded that strict quarantine rules are very effective in reducing the effective contact rate and hence the transmission of COVID-19. Ngonghala et al.^17^ proposed a mathematical model, by introducing non-pharmaceutical interventions such as social-distancing, contact-tracing and isolation to control and mitigate the burden of the pandemic. They have used the COVID-19 data to see the transmission dynamics in New York and the entire US. A COVID-19 model has been introduced by Sun and Wang^24^ to forecast the effect of implementing strong interventions in Heilongjiang province in order to minimize the total infectives.

Keeping in view the aforementioned papers, we have introduced a nonlinear mathematical model by providing vaccination to the population in order to reduce the burden of COVID-19 pandemic. The aim is also to minimize the infectives and the total financial burden of vaccination and treatment of COVID-19 disease in the society. The proposed model has been qualitatively and quantitatively analyzed following^19,23^.

The content of the paper is organized as follows: The second section begins with the formation of a nonlinear mathematical model along with the incorporation of vaccination. Equilibrium points have been determined along with computing the basic reproduction number in the third section. The fourth section captures the sensitivity index to determine the parameters that are having an immense impact on the reproduction number. An optimal control problem has been constructed in the fifth section, in order to reduce the infection prevalence together with the minimization of total financial burden on the society. Numerical simulations have been done in the sixth section to numerically validate the model with the real data of India. In the seventh section, the COVID-19 model has been discussed briefly.

## Model formation

In this section, a novel coronavirus model has been proposed and analyzed to study the transmission dynamics of COVID-19 disease among the population of India. To formulate the model, the total population denoted by *N*(*t*) depending on the time variable *t*,  is divided into eight mutually disjoint classes of population as defined in Table 1.

**Table 1:** The description of COVID-19 model state variables.

State variable	Description
*S*(*t*)	class of individuals susceptible to COVID-19 disease
*E*(*t*)	class of individuals exposed to COVID-19 (pre-symptomatically infected individuals)
*V*(*t*)	class of individuals vaccinated from COVID-19
$I_u(t)$	class of undetected infectives (majorly asymptomatic infectives)
$I_I(t)$	class of isolated infectives (includes both symptomatic and asymptomatic infectives)
$I_h(t)$	class of hospitalized infectives (those who requires intensive health care)
$R_u(t)$	class of unknown recovered individuals
$R_k(t)$	class of known recovered individuals
*D*(*t*)	class of individuals died from COVID-19

Therefore, the total population *N*(*t*) can be written as

$$\begin{aligned} N(t) = S(t)+E(t)+V(t)+I_u(t)+I_I(t)+I_h(t)+R_u(t)+R_k(t). \end{aligned}$$

To formulate the model following assumptions are taken into consideration:

- The population enters with a constant recruitment rate $\varPi$ into the class of susceptibles and dies with a natural death rate $\mu$.
- Each individual must enter into the exposed class after coming in contact with an infected individual before switching into other infected classes.
- Individuals recovered from COVID-19 attain short term immunity and will move to the class of susceptibles again.
- Population detected with COVID-19 infection will remain isolated even after the detection of disease as suggested by the health care authorities and start taking medical facilities if intensive care is required.
- In a highly populated country like India, the proportion of infected neonates is very less in comparison to the total infected cases of COVID-19 in India. Thus, we have not considered neonates born to infected mothers as infectives.

Susceptibles acquire COVID-19 infection and become exposed, due to effective contact with undetected infectives and hospitalized infectives at the rate $\beta$, where the force of infection, $\lambda$, is given as

1 $$\begin{aligned} \lambda =\frac{\beta }{N}(I_u(t)+ \eta I_h(t)), \end{aligned}$$

where $\eta <1$ is the modification parameter which accounts for the fact that hospitalized infectives are detected with the coronavirus disease and are under intensive care and thus follow more precautionary measures in relation to undetected individuals, which as a result, reduces the probability of acquiring infection from hospitalized infectives. Individuals exposed to COVID-19 may move to the class of vaccinated individuals after taking vaccination at the rate $m_1$. However, susceptibles may also start taking vaccination at the rate *m*. Individuals vaccinated from COVID-19 may not get permanent immunity and acquire COVID-19 infection by switching to the class of individuals exposed to COVID-19 at the rate $(1-\varepsilon )\lambda$, where $\varepsilon$ is the efficacy of vaccination. Exposed individuals with mild symptoms known as pre-symptomatically infected individuals remain under an incubation period for $5-10$ days. A fraction of newly infected individuals, who are not getting detected even after the completion of incubation period, will move into the class of undetected infectives at the rate $(1-\alpha ) k$. Undetected infectives may also progress to the class of isolated infectives by putting themselves into isolation or the quarantine centers set up by the government, after getting detected with the disease either by contact tracing or the generation of COVID-19 symptoms such as cold, high fever and difficulty in breathing at the rate $\sigma I_u$. Undetected infectives may also get recovered on their own from COVID-19 infection, on the basis of immunity level, and headway to the class of unknown recovered at the rate $\phi I_u$, from where they may again become susceptible at the rate $\frac{1}{\delta _2} R_u$. Infectives under isolation may also get recovered by themselves by taking a little medical assistance during isolation period and move to the class of known recovered at the rate $\tau I_I$. Individuals recovered from COVID-19 may not get permanent immunity and again move to the class of susceptibles at the rate $\frac{1}{\delta _1}R_k$. However, some infectives such as those with comorbidities and weakened immune system might require intensive medical care and headway to the class of hospitalized infectives, that is, $I_h(t)$ at the rate $\gamma I_I$. After getting recovered from COVID-19, hospitalized infectives enter into the class of known recovered at the rate $\psi I_h$. Undetected COVID-19 infectives, isolated infectives and hospitalized infectives who are dying due to COVID-19 move to the class of disease induced deaths at the rate $\mu _u, \mu _I$ and $\mu _h$, respectively.

**Figure 1:**
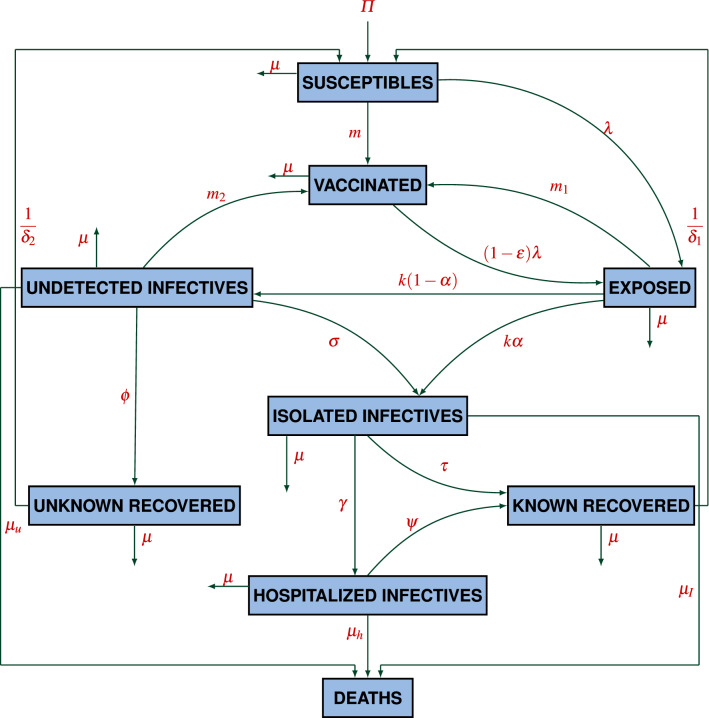
Schematic diagram describing the transmission of COVID-19.

Corresponding to the aforementioned assumptions, the variables and the parameters described in Tables 1 and 2, respectively, a non-linear mathematical model has been formulated on the basis of a schematic diagram as given in Fig. 1. The non-linear system of differential equations describing the model is given as

2 $$\begin{aligned}\frac{dS}{dt}&=\varPi -\lambda S-mS+\frac{1}{\delta _1}R_k+\frac{1}{\delta _2}R_u-\mu S\\ \frac{dE}{dt}&=\lambda S+\rho (1-\varepsilon ) \lambda V -m_1 E -k E- \mu E\\ \frac{dV}{dt}&=mS+m_1 E+m_2 I_u-\rho (1-\varepsilon ) \lambda V-\mu V\\ \frac{dI_u}{dt}&=(1-\alpha ) k E-m_2 I_u-\sigma I_u-\phi I_u-(\mu +\mu _u) I_u\\ \frac{dI_I}{dt}&=\alpha k E+\sigma I_u-\gamma I_I-\tau I_I- (\mu + \mu _I) I_I\\ \frac{dI_h}{dt}&=\gamma I_I- \psi I_h-(\mu +\mu _h) I_h\\ \frac{dR_u}{dt}&= \phi I_u-\frac{1}{\delta _2}R_u-\mu R_u\\ \frac{dR_k}{dt}&= \psi I_h+ \tau I_I-\frac{1}{\delta _1}R_k-\mu R_k, \end{aligned}$$

with the initial conditions given as

3 $$\begin{aligned} S(0) &=S_0\geqslant 0,\ E(0)= E_0 \geqslant 0,\ V(0)= V_0 \geqslant 0, \ I_u(0)=I_{u_0}\geqslant 0, \\ I_I(0)&=I_{I_0}\geqslant 0,\ I_h(0)=I_{h_0}\geqslant 0,\ R_u(0)=R_{u_0}\geqslant 0 \text{ and } R_k(0)=R_{k_0}\geqslant 0. \end{aligned}$$

**Table 2:** The description of parameters.

Parameter	Description
$\varPi$	Constant recruitment rate
$\beta$	Transmission rate for COVID-19
$\mu$	Natural death rate
$\mu _{u}$	Death rate of undetected infectives due to COVID-19
$\mu _I$	Death rate of isolated infectives due to COVID-19
$\mu _{h}$	Death rate of hospitalized infectives due to COVID-19
*k*	Incubation period for exposed individuals
$\varepsilon$	Efficacy of vaccination for COVID-19
*m*	Rate at which susceptibles attain vaccination against COVID-19
$m_1$	Rate at which exposed individuals attain vaccination against COVID-19
$m_2$	Rate at which undetected infectives attain vaccination against COVID-19
$\delta _1$	Period after which known recovered individuals become susceptibles
$\delta _2$	Period after which unknown recovered individuals become susceptibles
$\psi$	Recovery rate of hospitalized infectives
$\gamma$	Treatment rate at which isolated infectives get hospitalized
$\tau$	Rate at which isolated infectives get recovered themselves
$\alpha$	Fraction of exposed individuals detected from COVID-19
$\phi$	Recovery rate of undetected infectives
$\sigma$	Detection rate of undetected infectives through contact tracing
$\eta$	Modification parameter

### Basic properties of the model

All the variables $S(t),\ E(t),$ $\ V(t),\ I_u(t),\ I_I(t),\ I_h(t), \ R_u(t)$ and $R_k(t)$ forming the model system (2), denotes human sub-population, and hence are positive for all time $t \geqslant 0$^25^. Further, by adding the rate of change in all the sub-populations given by model system (2), we obtain

4 $$\begin{aligned} \frac{d N}{d t} =&\ \varPi -\mu N(t) -\mu _u I_u-\mu _I I_I-\mu _h I_h \leqslant \ \varPi -\mu N(t). \end{aligned}$$

We solve the above linear differential equation to obtain

$$\begin{aligned} N(t) \leqslant N(0) e^{-\mu t}+\frac{\varPi }{\mu }(1-e^{-\mu t}), \end{aligned}$$

from which it follows that $0 < N(t) \leqslant \frac{\varPi }{\mu }$, if $N(0) \leqslant \frac{\varPi }{\mu }$. Thus, the total population *N*(*t*) is bounded, which in turn, proves the boundedness of all the sub-populations of *N*(*t*). Therefore, without loss of generality, the following positively invariant region can be taken under consideration:

$$\begin{aligned} \varOmega =&\bigg \{(S,E,V,I_u,I_I,I_h,R_u,R_k)\in {\mathbb {R}}_+^{8} : N(t)\leqslant \frac{\varPi }{\mu }\bigg \}. \end{aligned}$$

In the model, a class of individuals died due to COVID-19 has been introduced and the corresponding differential equation for the death class is obtained as

5 $$\begin{aligned} \frac{d D}{dt} = \mu _u I_u + \mu _I I_I+\mu _h I_h. \end{aligned}$$

Thus, by solving equation (5) with respect to the time variable $t\ge 0$, the number of individuals died due to COVID-19 is obtained as

$$\begin{aligned} D(t) = D_0 +\mu _u \int \limits _0^{t} I_u d\xi +\mu _I \int \limits _0^{t} I_I d\xi +\mu _h \int \limits _0^{t} I_h d\xi . \end{aligned}$$

## Equilibrium points

There are two equilibrium points for the model system (2) that has been computed in the region $$\varOmega$$, namely, a disease-free equilibrium point $$P^0=(S^0,E^0,V^0,$$ $$I^0_u,I^0_I,I^0_h,R^0_u,R^0_k)$$, which represents the state when no COVID-19 infected individual is present in the population and an interior endemic equilibrium point $$P^*=(S^*,E^*,V^*,I^*_u,I^*_I,I^*_h,R^*_u,R^*_k)$$.

The components of both the equilibrium points can be obtained by solving the following simultaneous system of equations:

6 $$\begin{aligned} \varPi -\lambda S-mS+\frac{1}{\delta _1}R_k+\frac{1}{\delta _2}R_u-\mu S&=0\\ \lambda S+\rho (1-\varepsilon ) \lambda V -m_1 E -k_1 E- \mu E&=0\\ mS+m_1 E+m_2 I_u-\rho (1-\varepsilon ) \lambda V-\mu V&=0\\ (1-\alpha ) k E-m_2 I_u-\sigma I_u-\phi I_u-(\mu +\mu _u) I_u&=0\\ \alpha k E+\sigma I_u-\gamma I_I-\tau I_I- (\mu + \mu _I) I_I&=0\\ \gamma I_I- \psi I_h-(\mu +\mu _h) I_h&=0\\ \phi I_u-\frac{1}{\delta _2}R_u-\mu R_u&=0\\ \psi I_h+ \tau I_I-\frac{1}{\delta _1}R_k-\mu R_k&=0.\\ \end{aligned}$$

After solving for the uninfected components in the above system of equations, the disease-free equilibrium point is computed as $P^0=\left( \frac{\varPi }{m+\mu },0,\frac{\varPi m}{\mu (m+\mu )},0,0,0,0,0\right)$.

The basic reproduction number ${\mathcal {R}}_0$, is a threshold quantity, which counts the average number of secondary infectious cases produced by a single infected individual in an entirely susceptible population^9^. Mathematically, it can be computed using the matrices *F* and *V* corresponding to the new infection terms and the remaining transfer terms, respectively^32^ that are given as

7 $$\begin{aligned}F = \begin{bmatrix} 0 &{} \frac{\beta \left( \mu + \rho (1-\varepsilon )m \right) }{m+\mu } &{} 0 &{} \frac{\eta \beta \left( \mu + \rho (1-\varepsilon )m \right) }{m+\mu }\\ 0 &{} 0 &{} 0 &{} 0 \\ 0 &{} 0 &{} 0 &{} 0\\ 0 &{} 0 &{} 0 &{} 0\\ \end{bmatrix} \end{aligned}$$

8 $$\begin{aligned}V = \begin{bmatrix} m_1+ k +\mu &{} 0 &{} 0 &{} 0 \\ (1-\alpha )k &{} \phi +m_2+\sigma +\mu _u+\mu &{} 0 &{} 0\\ - \alpha k&{} -\sigma &{} \gamma +\tau +\mu _{I}+\mu &{} 0 \\ 0 &{} 0 &{} - \gamma &{} \psi +\mu _h +\mu \\ \end{bmatrix}. \end{aligned}$$

Therefore, the basic reproduction number is determined as

9 $$\begin{aligned} {\mathcal {R}}_0 = \frac{\beta k (\mu +m \rho (1-\varepsilon ))}{A_1 A_2 A_3 A_4 A_5} \left( (1-\alpha )(A_4 A_5+\eta \gamma \sigma )+A_3 \alpha \eta \gamma \right) , \end{aligned}$$

where

$$\begin{aligned} A_1= m+\mu ,\ \ A_2=k+m_1 +\mu , \ \ A_3=m_2+\sigma +\phi +\mu +\mu _u,\ \ A_4= \psi +\mu +\mu _h \ \text{ and } \ A_5=\gamma +\tau +\mu +\mu _I. \end{aligned}$$

### Stability analysis of the disease-free equilibrium point

To analyze the behavior of solution trajectories around the disease-free equilibrium point, the stability analysis is performed. We use the Lyapunov LaSalle’s Principle to prove the asymptotic stability of the disease-free equilibrium point.

#### Lemma 3.1

^11^ (Lyapunov LaSalle’s Principle) Let $\varOmega \subset B$ be a compact set that is positively invariant. Let $V: B \rightarrow {\mathbb {R}}$ be a continuously differentiable function and assume that ${\dot{V}}(x)\leqslant 0$ for all $x \in \varOmega$. Let *D* be the set of all points in $\varOmega$ where ${\dot{V}}(x) =0$. Let *S* be the largest invariant set in *D*. Then every solution starting in $\varOmega$ approaches *S* as $t \rightarrow \infty$, that is, for every $x(0) \in \varOmega$, $x(t) \rightarrow S$ as $t \rightarrow \infty$.

#### Theorem 3.2

The disease-free equilibrium point $P^0$ for the model system (2) is asymptotically stable, if ${\mathcal {R}}_0<1$ and is unstable for ${\mathcal {R}}_0>1$.

#### Proof

Let us consider a vector $Z=(E,I_u,I_I,I_h)^T$. We have to show that

$$\begin{aligned} \frac{dZ}{dt} \leqslant (F-V) Z, \end{aligned}$$

where the matrices *F* and *V* are given by equation (7) and (8), respectively. Clearly,

$$\begin{aligned} \frac{dZ}{dt}= \begin{bmatrix} \lambda S+\rho (1-\varepsilon ) \lambda V -m_1 E -k E- \mu E\\ (1-\alpha ) k E-m_2 I_u-\sigma I_u-\phi I_u-(\mu +\mu _u) I_u\\ \alpha k E+\sigma I_u-\gamma I_I-\tau I_I- (\mu + \mu _I) I_I\\ \gamma I_I- \psi I_h-(\mu +\mu _h) I_h \end{bmatrix} \leqslant (F-V)Z. \end{aligned}$$

Now, we define $X=(0, \beta , 0, \eta \beta )$. It is straightforward to verify that $X (V^{-1} F)= {\mathcal {R}}_0 X$. Therefore, *X* is a left eigenvector of $V^{-1} F$ associated with the eigenvalue ${\mathcal {R}}_0.$ Consider a Lyapunov function of the form

$$\begin{aligned} {\mathcal {L}}=X (V^{-1} Z). \end{aligned}$$

By differentiating ${\mathcal {L}}$ with respect to time, we get

$$\begin{aligned} \frac{d {\mathcal {L}}}{dt}=&\ X V^{-1} \frac{dZ}{dt} \leqslant \ X V^{-1}(F-V)Z \ = X({\mathcal {R}}_0-1) Z. \end{aligned}$$

If ${\mathcal {R}}_0<1$, then $X({\mathcal {R}}_0-1) Z < 0$. Therefore, the equality $\frac{d {\mathcal {L}}}{dt}=0$ gives $X Z=0$. Thus, we obtain

10 $$\begin{aligned} \beta (I_u+\eta I_h)=0. \end{aligned}$$

By using equation (6) and (10) we get, $E=I_u=I_I=I_h=R_u=R_k=0,$ $S=\frac{\varPi }{m+\mu }$ and $V=\frac{\varPi m}{\mu (m+\mu )}$. Thus, $\frac{d {\mathcal {L}}}{dt}=0$ for $P^0=\left( \frac{\varPi }{m+\mu },0,\frac{\varPi m}{\mu (m+\mu )},0,0,0,0,0\right)$. Therefore, the largest invariant set on which $\frac{d {\mathcal {L}}}{dt}=0$ is singleton $\{ P^0\}$. Hence, by the Lyapunov LaSalle’s Principle, it can be conclude that the disease-free equilibrium point $P^0$ is asymptotically stable when ${\mathcal {R}}_0<1$. However, if ${\mathcal {R}}_0>1$, we can observe $\frac{d {\mathcal {L}}}{dt}>0$ in a neighborhood of the disease-free equilibrium point in $\varOmega$. Thus, by Lyapunov stability theory the disease-free equilibrium point becomes unstable if ${\mathcal {R}}_0>1$. $\square$

### Endemic equilibrium point

The solution of the simultaneous system of equations (6), gives the components of the endemic equilibrium point $P^*=(S^*,E^*,V^*,I_u^*,I_I^*,I_h^*,R_u^*,R_k^*)$ as

11 $$\begin{aligned} S^*=&\ \frac{\varPi }{S_1} +\left( \frac{\psi \gamma +\tau A_4}{\gamma S_1 S_2}\right) I_h^* + \frac{\phi (1-\alpha )A_4 A_5}{\gamma S_1 S_3 S_4}I_h^* \\ E^*=&\ \frac{A_3 A_4 A_5}{\gamma S_2 S_4}I_h^*\\ V^*=&\ \frac{m\varPi }{S_1 S_5}+\left( \frac{m(\psi \gamma +\tau A_4)}{\gamma S_1 S_2 S_5} + \frac{m\phi (1-\alpha ) A_4 A_5}{\gamma S_1 S_3 S_4 S_5}\right) I_h^* +\left( \frac{m_1 A_3 A_4 A_5}{\gamma k_1 S_4 S_5} + \frac{m_2 (1-\alpha ) A_4 A_5}{\gamma S_4 S_5}\right) I_h^* \\ I_u^*=&\ \frac{(1-\alpha )A_4 A_5}{\gamma S_4}I_h^*\\ I_I^*=&\ \frac{A_4}{\gamma } I_h^* \\ I_h^*=&\ \frac{ \varPi \gamma k S_2 S_3 S_4(\rho (1-\varepsilon )(\lambda ^*+m)+\mu ) }{{\mathcal {I}}_1 -{\mathcal {I}}_2}\\ R^*_u=&\ \frac{\phi \delta _2(1-\alpha )A_4 A_5}{\gamma S_2 S_4}I_h^*\\ R^*_k=&\ \frac{\delta _1\left( \psi \gamma +\tau A_4 \right) }{\gamma S_2}I_h^*, \end{aligned}$$

where

$$\begin{aligned} S_1 &= \lambda ^* +m+\mu ,\ S_2 = 1+\mu \delta _1,\ S_3 = 1+\mu \delta _2,\ S_4 = \sigma -\alpha \sigma +\alpha A_3,\ S_5 =\lambda ^* \rho (1-\varepsilon )+\mu ,\\ {\mathcal {I}}_1 &= S_1 S_2 S_3 A_4 A_5\left( A_3 (\rho (1-\varepsilon )\lambda ^*(k+\mu ) +\mu A_2) +\rho (1-\varepsilon )m_2 k(1-\alpha )\right) \quad {\text{ and }} \\ {\mathcal {I}}_2 &= ((\psi \gamma +\tau A_4) S_3 S_4 +\phi S_2 A_4 A_5 (1-\alpha )) (\rho (1-\varepsilon )(\lambda ^*+m)+\mu).\end{aligned}$$

The force of infection $\lambda ^*$, appearing in the components of endemic equilibrium point can be determined by using the following expression

12 $$\begin{aligned} \lambda ^*= \frac{\beta }{N}(I^*_u+\eta I^*_h). \end{aligned}$$

**Table 3:** Parameters value for the numerical simulations.

Parameter	Value	Source	Parameter	Value	Source
$\varPi$	50,000 day $^{-1}$	Assumed	$\varepsilon$	0.8	Estimated
$\beta$	1.12 day $^{-1}$	Assumed	$\sigma$	0.45 day $^{-1}$	Data fitted
$\mu$	0.000039 day $^{-1}$	Estimated	$\phi$	0.9 day $^{-1}$	Data fitted
$\mu _{u}$	0.0001 day $^{-1}$	Assumed	$\psi$	1/14.7 day $^{-1}$	Estimated
$\mu _I$	0.000162 day $^{-1}$	Estimated	$\gamma$	0.3 day $^{-1}$	Estimated
$\mu _{h}$	0.002528 day $^{-1}$	Estimated	$\tau$	0.9 day $^{-1}$	Estimated
*k*	1/8 day $^{-1}$	Estimated	$\delta _1$	40	Assumed
$\alpha$	0.2	Data fitted	$\delta _2$	40	Assumed
$\eta$	0.7	Assumed	*m*	0.00016 day $^{-1}$	Estimated
$m_1$	0.00005 day $^{-1}$	Estimated	$m_2$	0.00005 day $^{-1}$	Estimated

## Sensitivity analysis

The threshold quantity ${\mathcal {R}}_0$ plays a vital role to understand the transmission of a disease into a community. In the proposed non-linear mathematical model, the aforementioned parameters have different levels of impact on ${\mathcal {R}}_0$. This section consists of the sensitivity index of the basic reproduction number ${\mathcal {R}}_0$ with respect to various model parameters. We investigate the significance of various parameters that have immense impact such as reduction in deaths due to COVID-19 infection. In relation to this, we study the normalized forward sensitivity index of ${\mathcal {R}}_0$ corresponding to the various parameters. If the basic reproduction number is differentiable with respect to a parameter, then the sensitivity index can also be obtained by using the partial derivative. In the model prediction, the vitality of various parameters can also be justified by the sensitivity analysis. The occurrence of errors on the basis of collected data and presumed values may not justify the significance of a mathematical model.

### Definition 4.1

^3^ Let, ${\mathcal {V}}$ be the dependent function and is differentiable with respect to parameter *p*, then the normalized forward sensitivity index is defined as:

13 $$\begin{aligned} \varUpsilon _p^{{\mathcal {V}}} := \frac{\partial {\mathcal {V}}}{\partial p} \frac{p}{{\mathcal {V}}}. \end{aligned}$$

The sensitivity index of ${\mathcal {R}}_0$ determines the parameters (given in (9)) having an immense impact on ${\mathcal {R}}_0$. According to the parameters value given in Table 3 the sensitivity index for the transmission rate $(\beta )$ is given as

$$\begin{aligned} \varUpsilon _\beta ^{R_0} :=&\ \frac{\partial {\mathcal {R}}_0}{\partial \beta } \frac{\beta }{{\mathcal {R}}_0} \\ =&\ +1. \end{aligned}$$

**Figure 2:**
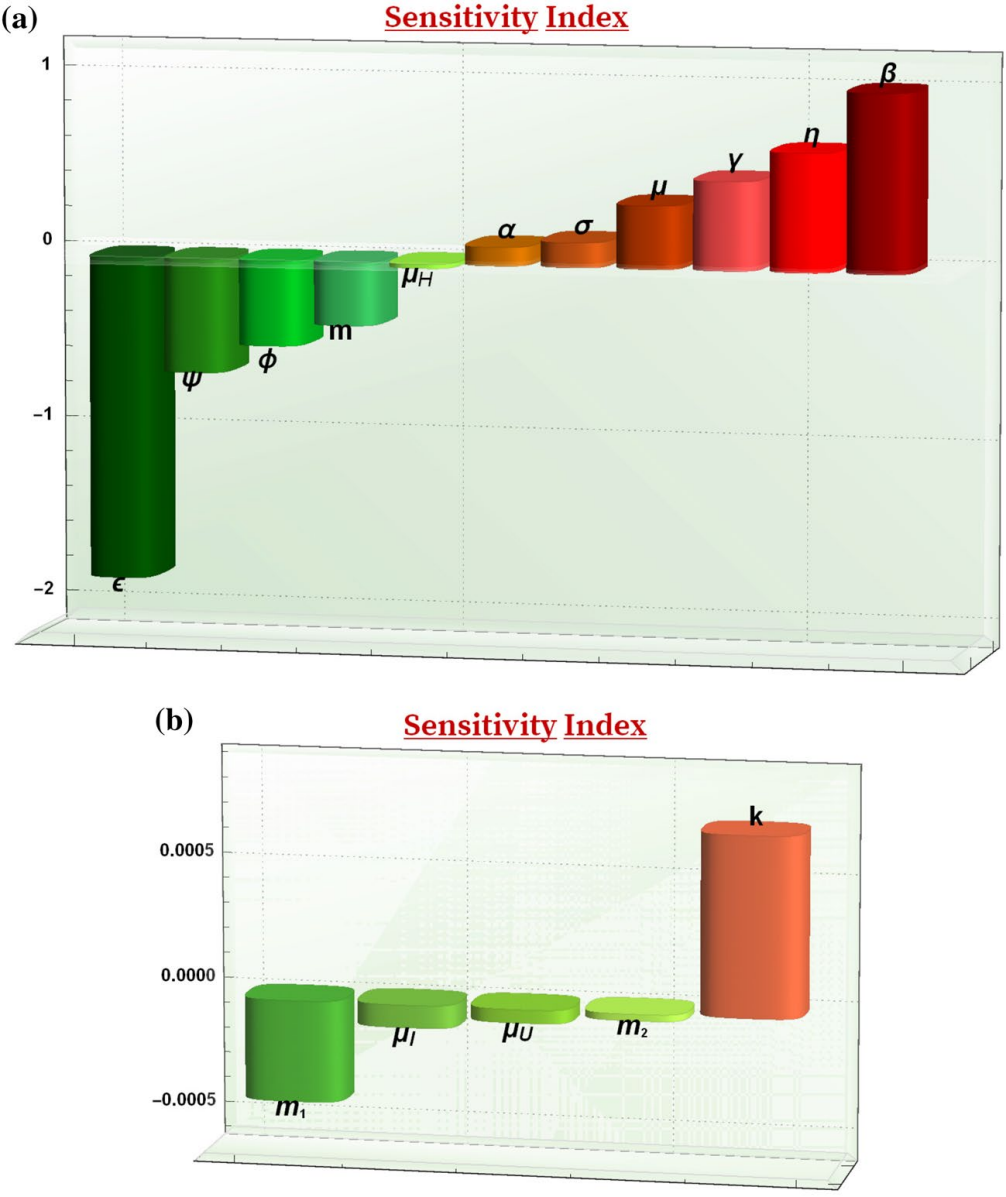
Graphs showing the sensitivity of the reproduction number with respect to all the parameters.

The relative impact of various parameters on the threshold quantity ${\mathcal {R}}_0$ is shown below in Fig. 2 and the following points are observed:

- $\varUpsilon _\beta ^{{\mathcal {R}}_0} =1$: The value 1 signifies that ${\mathcal {R}}_0$ is directly proportional to $\beta$. Therefore, the transmission rate must be essentially reduced by increasing the detection and isolation of infectives and strictly following precautionary measures by all the individuals.
- $\varUpsilon _\varepsilon ^{{\mathcal {R}}_0} =-1.80282$: From this value of sensitivity index of ${\mathcal {R}}_0$, it is observed that if $\varepsilon$ increases by 10% then ${\mathcal {R}}_0$ decreases by approximately 18%. That is, the efficacy of vaccination has a very high significance in the model. It signifies that the vaccinated individuals still need to follow the precautionary measures to control the transmission of infection as vaccinated individuals may also transmit the infection even without getting symptoms.
- $\varUpsilon _\eta ^{{\mathcal {R}}_0} =0.661243$: From this point, we can observe that ${\mathcal {R}}_0$ increases by 6.612% if $\eta$ increases by 10%. Therefore, the health care authorities are required to follow more precautionary measures while giving treatment to the hospitalized infectives.
- $\varUpsilon _m^{{\mathcal {R}}_0} =-0.353316$: This value of sensitivity index indicates that the threshold quantity ${\mathcal {R}}_0$ decreases by 3.53% whenever *m* increases by 10%. However, at this rate of vaccination the spread of COVID-19 disease infection may not be controlled and, thus the vaccination must be provided with high intensity in order to eradicate the disease.

From the sensitivity index, it is concluded that only carrying out the precautionary measures is not adequate enough to eradicate the disease. The transmission of the COVID-19 infection can be controlled by giving vaccination to the individuals on its maximal level on the basis of precedence. Also, to increase the efficacy of vaccination each vaccinated individual is also required to follow the precautionary measures to maintain the effect of vaccination as they may also infect others without getting the symptoms of COVID-19 disease.

## Optimal control problem

Modeling on an infectious disease may involve a high number of constant parameters, transmission effects and treatment costs. To make the model realistic, one requires the optimal control interventions on the level of infection and management costs to optimize the disease burden as much as possible. This model investigates the transmission dynamics of COVID-19 infection in India. Since, India is a highly populated country therefore the transmission of COVID-19 infection is higher and hence, it involves the high costs of management. In this paper, optimal control strategies are applied to minimize the number of infectives, the treatment cost on individuals and financial burden on the health care authorities. It has been that the optimal control strategies play a vital role in optimizing the infectious disease problems^25^. In this section, we transform the constant parameters system of differential equations (2) into a system of differential equations with time dependent control functions. We introduce the control functions $u_1(t),u_2(t),u_3(t)$ and $u_4(t)$ by replacing the constant vaccination parameters $m,m_1,m_2$ and the intensive care treatment rate $\gamma$, respectively. The control function $u_1(t),u_2(t)$ and $u_3(t)$ represent the time dependent vaccination rate to the susceptibles, exposed individuals and undetected infectives from COVID-19. Also, $u_4(t)$ measures the time dependent rate at which isolated infectives attain intensive treatment.

The optimal control strategies are applied to solve the problem by using Pontryagin’s maximum principle^20^. According to this principle, we identify the intensity of optimal interventions and ideal timing to minimize the COVID-19 infected individuals along with reducing the cost as much as possible for the finite time interval. Thus, the optimal control functions $u_1(t),u_2(t),u_3(t)$ and $u_4(t)$ will be chosen in such a way that the state variables and the control functions minimize the objective functional defined as

$$\begin{aligned} J(u_1,u_2,u_3,u_4)=\int \limits _0^ {t_f}&\Big (W_1 I_u+W_2 I_I+W_3 E+ W_4(\mu _u I_u+\mu _I I_I+\mu _h I_h) + \frac{B_1}{2} u_1^2+\frac{B_2}{2} u_2^2+ \frac{B_3}{2} u_3^2+\frac{B_4}{2} u_4^2 \Big ) dt, \end{aligned}$$

where $B_1,\ B_2,\ B_3$ and $B_4$ denote the cost coefficients and the corresponding terms $\frac{B_1}{2}u_1^2$, $\frac{B_2}{2}u_2^2$ and $\frac{B_3}{2}u_3^2$ describe the vaccination costs and the term $\frac{B_4}{2}u_4^2$ indicates the treatment cost. Also, the balancing factors are represented by the coefficients $W_1, W_2, W_3$ and $W_4$ indicating the significance of one type of intervention over the others. Define, the set of admissible control functions as

14 $$\begin{aligned} \varSigma =&\{(u_1,u_2,u_3,u_4): \text { measurable and } 0\leqslant u_1,u_2,u_3,u_4 \leqslant 1 \text { for } t \in [0, t_f]\}, \end{aligned}$$

where $t_f$ denotes the final time of study period. The objective is to determine the optimal control values $u_1^*, u_2^*, u_3^*$ and $u_4^*$ that satisfy the conditions in (14) and

15 $$\begin{aligned} J(u_1^*,u_2^*,u_3^*,u_4^*) = \underset{\varSigma }{\min } \ J(u_1,u_2,u_3,u_4). \end{aligned}$$

We first verify the existence conditions given by Fleming and Rishel^7^ for the optimal control function $u^*=(u_1^*,u_2^*,u_3^*,u_4^*)$ and solution trajectories for the state variables:

1. The solution set for the model system (2) must be non-empty corresponding to the admissible control functions in $\varSigma$. Since, $0 \le N(t)\le \frac{\varPi }{\mu }$ and $N(t) = S(t)+E(t)+V(t)+I_u(t)+I_I(t)+I_h(t)+R_u(t)+R_k(t).$ Therefore, all the state equations are continuous and bounded.
  Further, since all the partial derivatives with respect to state variables are bounded, thus, the Lipschitz condition is satisfied. Therefore, by the Picard-Lindelof’s theorem^4^, the solution set for the initial value problem (2)–(3) along with the control functions in $\varSigma$ is non-empty.
2. The system of differential equations in (2) can be expressed as a linear function of control variables with the coefficients representing the functions of time and state variables as
  16 $$\begin{aligned} h_i=a(t,x_i)+b(t,x_i)u_j. \end{aligned}$$
  Here, $x_i's$ denote the state variables for $i=1,2, \dots , 8$, whereas, for $j=1,2,3,4$, $u_j's$ denotes the associated control variable.
3. The Lagrangian function in the objective functional *J* of the problem, is given as
  $$\begin{aligned} {\mathcal {L}}=W_1 I_u+W_2 I_I+W_3 E+W_4(\mu _u I_u+\mu _I I_I+\mu _h I_h)+ \frac{B_1}{2} u_1^2+\frac{B_2}{2} u_2^2+\frac{B_3}{2} u_3^2+\frac{B_4}{2} u_4^2. \end{aligned}$$
  The Lagrangian function ${\mathcal {L}}$ must be convex on $\varSigma$ and satisfies the relation
  17 $$\begin{aligned} {\mathcal {L}}(x,t,u)\geqslant \zeta _1 \mid u \mid ^\xi - \ \zeta _2 , \text{ where } \zeta _1> 0 \text{ and } \xi >1. \end{aligned}$$
  Clearly, the function ${\mathcal {L}}$ is convex on $\varSigma$ as it is a quadratic function of $u=(u_1,u_2,u_3,u_4)$. Also, since $B_4 u_4^2 \leqslant B_4 \ \text{ as } u_4 \in [0,1]$, therefore, the bound on ${\mathcal {L}}$ can be proved as
  $$\begin{aligned} {\mathcal {L}}(x,t,u)>&\ \frac{B_1}{2} u_1^2+\frac{B_2}{2} u_2^2+\frac{B_3}{2} u_3^2+\frac{B_4}{2} u_4^2\\ \geqslant&\ \frac{B_1}{2} u_1^2+\frac{B_2}{2} u_2^2+\frac{B_3}{2} u_3^2+\frac{B_4}{2} u_4^2-\frac{B_4}{2}\\ \geqslant&\ min\left( \frac{B_1}{2},\frac{B_2}{2},\frac{B_3}{2},\frac{B_4}{2}\right) (u_1^2+u_2^2+u_3^2+u_4^2)- \frac{B_4}{2}\\ =&\ min\left( \frac{B_1}{2},\frac{B_2}{2},\frac{B_3}{2},\frac{B_4}{2} \right) \mid u_1,u_2,u_3,u_4\mid ^2- \frac{B_4}{2}\\ =& \ \zeta _1 \mid u_1,u_2,u_3,u_4\mid ^{\xi } - \ \zeta _2, \end{aligned}$$
  where, $\zeta _1=min\left( \frac{B_1}{2},\frac{B_2}{2},\frac{B_3}{2},\frac{B_4}{2} \right)> 0, \ \zeta _2=\frac{B_4}{2} \text{ and } \xi >1.$ Therefore, the Lagrangian function ${\mathcal {L}}$ satisfies the relation in equation (17).

Thus, all the conditions of Fleming and Rishel^7^ are verified, and hence, we have proved the following result.

### Theorem 5.1

There exist an optimal control $u^*=(u_1^*,u_2^*,u_3^*,u_4^*)$ and the corresponding solution trajectories $S^*, E^*, V^*, I^*_u,$ $I^*_s, I^*_h, R^*_u \text{ and } R^*_k$ for the initial value problem (2)–(3) that minimizes $J(u_1,u_2,u_3,u_4)$ over $\varSigma$.

### Characterization of optimal control

Consider the initial value problem (2)–(3) with the objective functional *J*. We use the Pontryagin’s maximum principle which transforms the problem of minimizing the objective functional *J* into minimizing the Hamiltonian function ${\mathcal {H}}$ defined as

$$\begin{aligned} {\mathcal {H}}(x,u,\lambda _i) =&\ {\mathcal {L}}+ \sum _{i=1}^{8} \lambda _i h_i. \end{aligned}$$

Here, the term $x=(S,E,V,I_u,I_I,I_h,R_u,R_k)$ represent the vector of state variables and each $h_i$ represents the right hand side of the *i*th equation of state variables for the model system (2). To track the changes in the objective functional corresponding to the state variables, we utilize the adjoint variables that satisfy

18 $$\begin{aligned} \frac{d\lambda _i}{dt} = -\frac{\partial {\mathcal {H}}}{\partial x}, \end{aligned}$$

where $x=(S,E,V,I_u,I_I,I_h,R_u,R_k)$, together with the transversality conditions given as

19 $$\begin{aligned} \lambda _1(t_f)=\lambda _2(t_f)=\lambda _4(t_f)=\lambda _5(t_f)=\lambda _6(t_f)=\lambda _6(t_f)=\lambda _7(t_f)=\lambda _8(t_f)=0. \end{aligned}$$

#### Optimality conditions

Differentiation of the Hamiltonian function partially with respect to $u_1,u_2,u_3$ and $u_4$ and substituting $\frac{\partial {\mathcal {H}}}{\partial u} = 0$, gives

$$\begin{aligned} {\tilde{u}}_1=\frac{(\lambda _1-\lambda _3)}{B_1} S,&\ \ \ \ \ {\tilde{u}}_2=\frac{(\lambda _2-\lambda _3)}{B_2} E,\\ {\tilde{u}}_3=\frac{(\lambda _4-\lambda _3)}{B_3} I_u,&\ \ \ \ \ {\tilde{u}}_4=\frac{(\lambda _5-\lambda _7)}{B_4} I_I. \end{aligned}$$

In order to minimize the optimal controls $u_1,u_2,u_3$ and $u_4$ over $\varSigma$, the control parameters $u^*_1,u^*_2,u^*_3$ and $u^*_4$ must be bounded between 0 and 1. Thus, to control the transmission of COVID-19 infection the optimal control functions for the detection and treatment at time *t* are obtained as

20 $$\begin{aligned} u_1^* &= max\left( 0, min\left( 1, \frac{(\lambda _1-\lambda _3)}{B_1} S \right) \right) , \\ u_2^* &= max\left( 0, min\left( 1, \frac{(\lambda _2-\lambda _3)}{B_2} E \right) \right) , \\ u_3^* &= max\left( 0, min\left( 1, \frac{(\lambda _4-\lambda _3)}{B_3} I_u \right) \right) , \\ u_4^* &= max\left( 0, min\left( 1, \frac{(\lambda _5-\lambda _7)}{B_4} I_I \right) \right). \end{aligned}$$

## Numerical simulations

Corresponding to the epidemiological model of COVID-19 infection given by (2)–(3), the numerical simulations are performed on the basis of parameters value as given in Table 3 that are estimated by the data taken from the Ministry of Health and Family Welfare of India^15^, WHO^34,36^ and the published literature^14,17^. For the month of March/2021, the real data of India^15^ is taken under the consideration, and therefore, the initial values for the state variables are chosen as $S(0)=1,300,000,000, \ E(0)=400,000, \ V(0)=8,641,002, \ I_u(0)=560,000,$ $I_I(0)=160,293,\ I_h(0)=10,000, \ R_u(0)=7,000,000, \ R_k(0)=10,588,795$.

For the model (2), we have assumed the constant recruitment rate $\varPi = 50,000$ as the average number of births in India ranges between $45,000-70,000$ $\hbox {day}^{-1}$. Also, the natural death rate is evaluated at $\mu = 3.9 \times 10^{-5}\ \text{ day}^{-1}\ =\frac{1}{69.3} \times \frac{1}{365}\ \hbox {day}^{-1}$ as the average life expectancy of people in India is 69.3 years. According to WHO^35^, the average incubation period for the individuals progressing from the class of exposed to the infected classes is 8 days, which assisted us to choose $k=1/8$. Corresponding to the model, the transmission rate $\beta$ for the time duration March/01/2021 to March/25/2021 is assumed to be $1.12\ \hbox {day}^{-1}$. The relative infectiousness of hospitalized infectives is assumed to be $\eta =0.7$ as they infect at comparatively lesser rate than undetected infectives. Since, only 20–30% infected individuals require intensive medical treatment, therefore, $\gamma$ is assumed to be 0.3 day$^{-1}$. According to the report, the efficacy of vaccination is 81%, therefore, we have chosen $\varepsilon =0.8$. Also, the individuals getting recovered from COVID-19 may acquire immunity for approximately 40 days and then again become susceptible which justifies choosing $\delta _1=40$ and $\delta _2=40$. For the time duration March/01 to March/25/2021, the average number of daily active cases were estimated to be 162,214^6^ and daily average deaths due to COVID-19 infection were 103^6^. Since, hospitalized infectives are more prone to get dead due to COVID-19 because of the presence of other comorbidities, more than 70% of the total deaths due to COVID-19 are observed in hospitalized infectives. Therefore, we have subdivided the total deaths into 82 and 21 of hospitalized infectives and isolated infectives, respectively. Correspondingly, the daily actively infectives are categorized into the daily number of isolated infectives and hospitalized infectives as 129, 772 and 32, 442, respectively. Thus, the estimated case fatality rate during the hospitalization and isolation are $\mu _h = \frac{82}{32442} = 0.002528$ and $\mu _I = \frac{21}{129772} =0.000162$ $\hbox {day}^{-1}$. The death rate of undetected infectives is assumed to be $\mu _u = 0.0001$ $\hbox {day}^{-1}$. As reported by Ferguson et al.^8^, the infectives spend around 8 days in hospitalization and to monitor the regeneration of symptoms they remain under the isolation period for $4-6$. Therefore, $\psi$ is estimated at $\frac{1}{14.7}$ $\hbox {day}^{-1}$. In India, the recovery rate of infected individuals is more than 90%, therefore, we have estimated $\tau = 0.9$. The remaining model parameters values are estimated by data fitting and are given as $\alpha =0.2$, $\sigma = 0.45$, $\phi = 0.9$.

**Figure 3:**
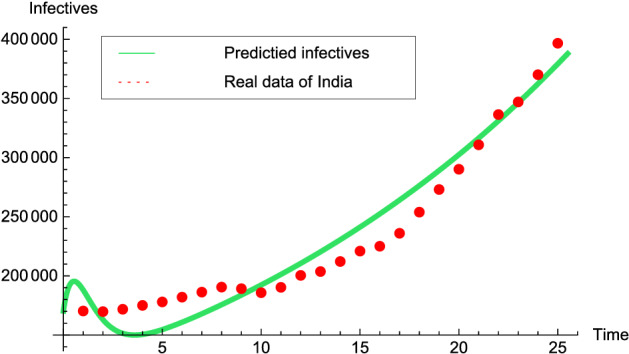
Graph illustrating the validation of model system to the real data of India for the time interval March/01-March/25/2021: Red dots and Green solid line depict the real data and predicted trajectory, respectively, for the model system (2).

To numerically simulate the model, we are initializing by validating the estimated data for the model. Correspondingly, the predicted number of infectives are compared to the real data of India. In Fig. 3, we can visualize the prevalence of COVID-19 infection in India for the time period March/01 to March/25/2021.

**Figure 4:**
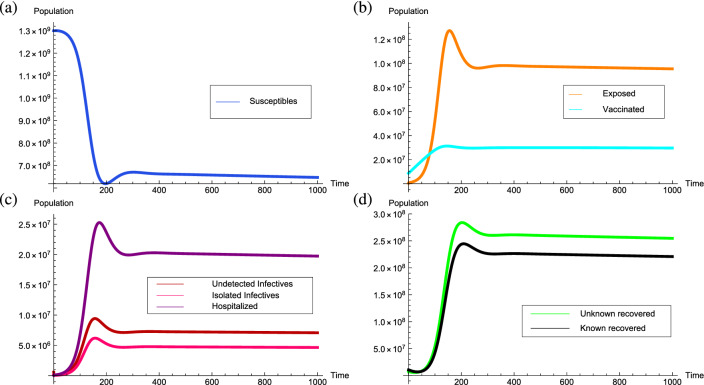
Graphs depicting the stability of the equilibrium point $P_2= (3.19754\times 10^8, 4.72041\times 10^7, 1.4516\times 10^7,3.49611\times 10^6,2.29408\times 10^6,$ $1.25664\times 10^8,9.74901\times 10^6, 1.08945\times 10^8)$ for ${\mathcal {R}}_0=0.698428<1$.

The behavior of the equilibrium points is numerically determined corresponding to the parameters value estimated in Table 3. Accordingly, the estimated threshold quantity is ${\mathcal {R}}_0=0.698428$ which is less than unity and the transmission rate is $\beta =1.12$ with the disease-free equilibrium point $P^0=(2.51256 \times 10^8, 0, 1.0308 \times 10^9, 0, 0, 0, 0, 0)$. Corresponding to the model, for ${\mathcal {R}}_0=0.698428$ two endemic equilibrium points come into existence. The point $P_1= (4.91813\times 10^8, 1.67406\times 10^6, 7.56626\times 10^8, 123,987, 813,57.6, 4.45657\times 10^6, 345,741, 3.86364\times 10^6)$ is an unstable endemic equilibrium point, whereas, the endemic equilibrium point $P_2= (3.19754\times 10^8, 4.72041\times 10^7, 1.4516\times 10^7, 3.49611\times 10^6, 2.29408\times 10^6, 1.25664\times 10^8, 9.74901\times 10^6, 1.08945\times 10^8)$ is stable. For the above initial conditions, if ${\mathcal {R}}_0=0.698428 < 1$ the solution trajectories approach to the endemic equilibrium point $P_2$ which justifies its local asymptotic stability and can be visualized in Fig. 4.

**Figure 5:**
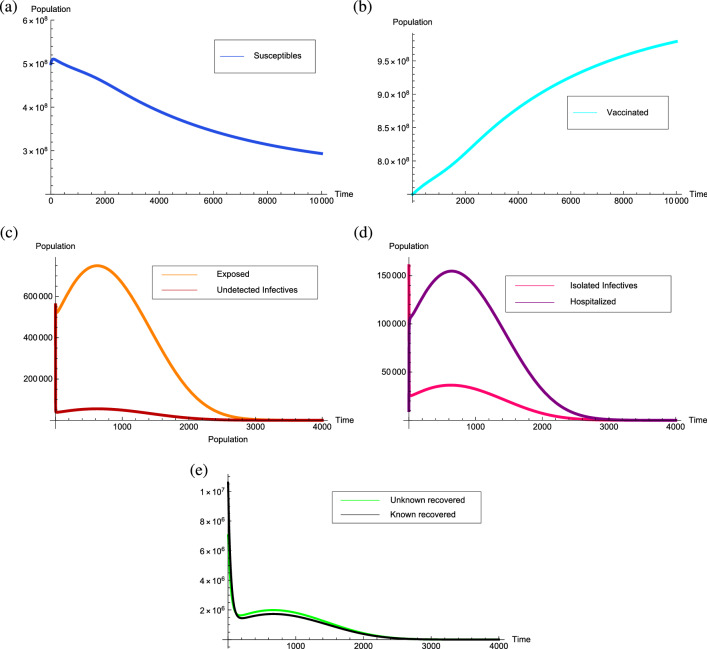
Graphs depicting the stability of the equilibrium point $P^0=(2.51256 \times 10^8, 0, 1.0308 \times 10^9, 0, 0, 0, 0, 0)$ for ${\mathcal {R}}_0=0.698428<1$.

From the Fig. 5, for ${\mathcal {R}}_0$ with less than unity if the initial conditions for the susceptibles and vaccinated individuals changes to $5 \times 10^8$ and $7.5 \times 10^8$, respectively, then the number of susceptibles and vaccinated individuals approach towards their respective components of the disease-free equilibrium point, whereas, the solution trajectories corresponding to all other classes approach towards zero in approximately 3000 days. Thus, it is observed that only the precautionary measures such as using the face masks and isolation of infectives are not sufficient enough to eradicate the disease from the population even when the reproduction number reduces below the unity. Thus, the vaccination rate must be increased especially for the individuals that are suffering from other immune based comorbidities and health care workers that reduces the reproduction number below unity and aid to eradicate the COVID-19 disease from the population.

**Figure 6:**
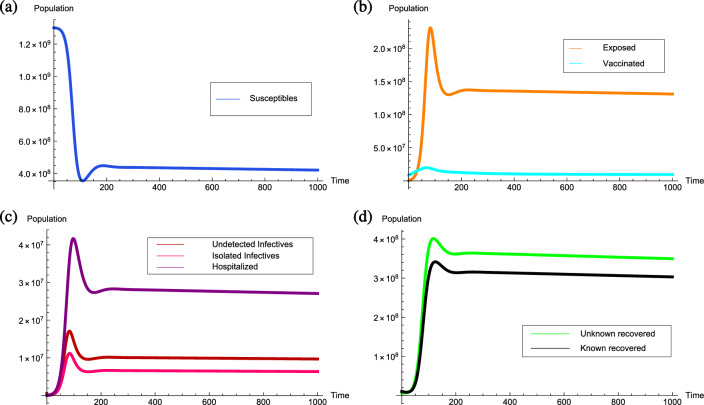
Graphs illustrating the COVID-19 infection prevalence together with the local stability of the endemic equilibrium point $P^*=(1.76186 \times 10^8,5.48786 \times 10^7,3.99376 \times 10^6,4.06451 \times 10^6, 2.66705 \times 10^6,$ $1.1334\times 10^7,1.46094\times 10^8,1.26657 \times 10^8)$ when the corresponding reproduction number for COVID-19 is greater than unity.

If the transmission rate $\beta$ reaches to 1.7, the threshold quantity will be ${\mathcal {R}}_0=1.06011>1$, then the unique point $P^*=(1.76186 \times 10^8,5.48786 \times 10^7,3.99376$ $\times 10^6,4.06451 \times 10^6, 2.66705 \times 10^6, 1.1334\times 10^7,1.46094\times 10^8,1.26657 \times 10^8)$ becomes endemic equilibrium point and locally asymptotically stable. It can be clearly observed from Fig. 6 that due to rapid increment in the number of infectives the number of susceptibles decreases with the higher rate and then starts increasing gradually. Therefore, the number of known and unknown recovered individuals keep on increasing with the significant rate.

**Figure 7:**
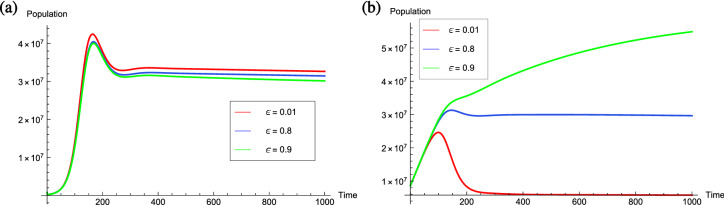
Graphs illustrating the influence of the efficacy of vaccination among individuals. The graphs are plotted by varying the value of $\varepsilon$ from 0.01 to 0.9 for; (**a**) Total infectives (**b**) Vaccinated individuals.

The COVID-19 vaccines were introduced worldwide several months ago. However, according to the World Health Organization it will be earlier to determine the curability from COVID-19 infection. Since, the efficacy of COVID-19 vaccine appear to be high yet no vaccine has been introduced to provide 100% protection. As a result, there may be a small percentage of people who do not develop protection as expected after the introduction COVID-19 vaccination. In addition to the vaccine’s specific characteristics, several factors such as a person’s age, the underlying health conditions or previous exposure to COVID-19 may have an impact on the vaccine’s effectiveness. The vaccine’s effectiveness can also be affected by prevention measures taken by the vaccinated individuals. A vaccine can be effective when there is a strong and healthy immune system, however, alcohol is believed to suppress the immune functioning during the vaccination time. Thus, it is advisable by the health care authorities for the vaccinated individuals to avoid the consumption of alcohol for at least 45 days after the vaccination. Also, the vaccinated individuals must follow all the precautionary measures as followed previously such as social distancing, wearing masks and avoid large gatherings. Since, all such precautionary measures play a vital role in increasing the effectiveness of vaccine. By keeping all these practices in concern, Fig. 7 shows the level of efficacy of vaccination on the infected and vaccinated individuals by varying the value of $\varepsilon$ from 0.01 to 0.9. From Fig. 7(a), it can be observed that infectives start decreasing if the efficacy of vaccination increases. However, the number of vaccinated individuals increases if the efficacy of vaccination increases. From the Fig. 7(a), (b), it can be visualized that it is necessary for everyone who have received the COVID-19 vaccine to follow the appropriate COVID-19 precautionary measures such as mask wearing, hand sanitization social distancing and work from home to increase the vaccination process more effective.

### Analysis of optimal controls

Now we shall carry out the numerical simulations to demonstrate the impact of optimal controls on the dynamics of COVID-19 by considering the parameters value as given in Table 3. As of March/25/2021, the number of actively infected cases in India were around $422,592$, whereas the recovered population has reached $11,292,838$^15^. In view of this the initial conditions are chosen as $S(0)=1,300,000,000, \ E(0)=700,000, \ V(0)=55,504,440, \ I_u(0)= 750,000,\ I_I(0)=380,333, I_h(0)=42,259, R_u(0)=8,000,000 { \text{ and } } R_k(0)=11,292,838$.

The optimal control profiles have been numerically interpreted for the time interval March/25/2021 to April/25/2021, therefore, the final time has been set to $t_f=30$ days. The fourth order Runge-Kutta iterative scheme is used to estimate the results, beginning with the initial guess for the control variables over the interval $[0,t_f]$. To numerically solve the system, we solve the state equations satisfying

$$\begin{aligned} \frac{dx}{dt} = -\frac{\partial {\mathcal {H}}}{\partial \lambda _i} \end{aligned}$$

together with the adjoint equations given by (18) and the control characterizations determined by equation (20). The state variables are computed by solving the system of state equations using the forward fourth order Runge-Kutta scheme. The adjoint equations are then numerically solved by applying backward Runge-Kutta technique with the given transversality conditions. The process continues and the control variables are updated at each iteration by substituting the new values of state and adjoint variables until the convergence occurs.

**Figure 8:**
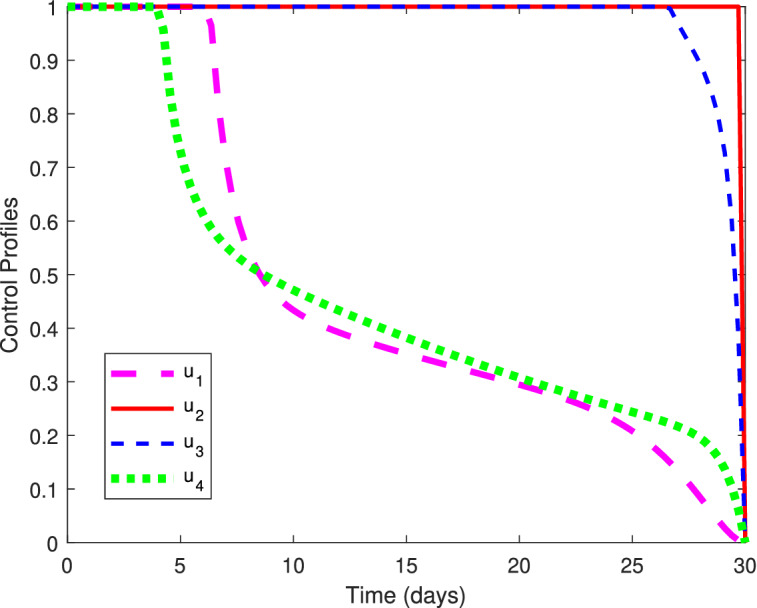
Illustration of optimal control profiles $u_1^*(t), u_2^*(t), u_3^*(t)$ and $u_4^*(t)$ for $B_1= B_2 = B_3=250$ and $B_4=1000$.

**Figure 9:**
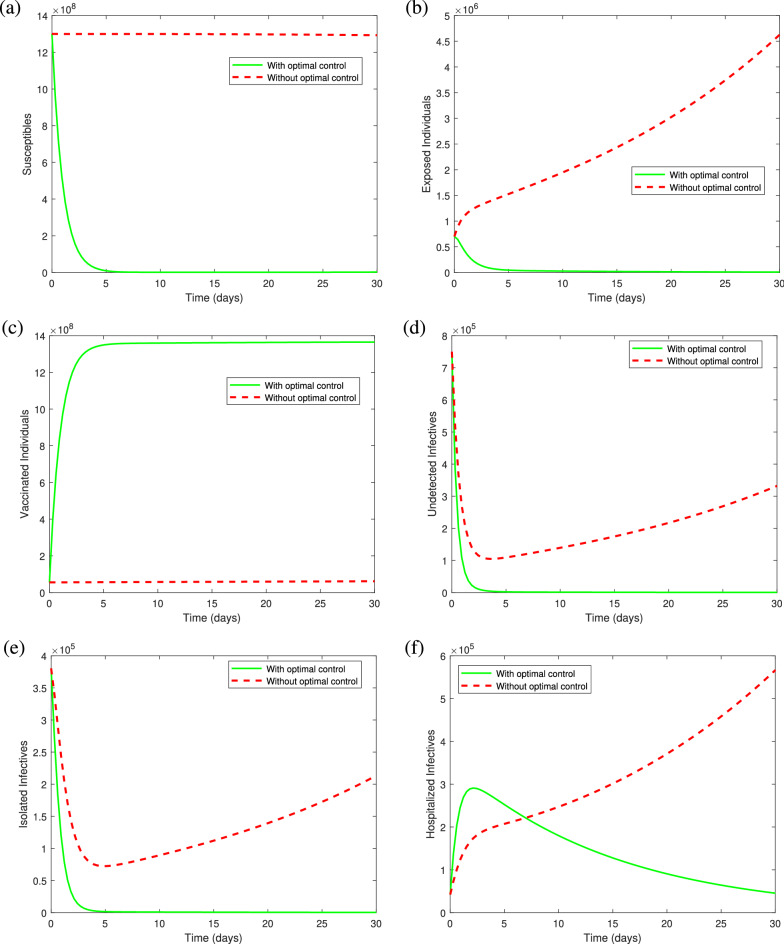
Graphs illustrating the solution trajectories against time (days) from March/25/2021 to April/25/2021 with and without optimal controls $u_1^*(t), u_2^*(t),$ $u_3^*(t)$ and $u_4^*(t)$: (**a**) Susceptibles (**b**) Exposed individuals (**c**) Vaccinated individuals (**d**) Undetected infectives (**e**) Isolated infectives (**f**) Hospitalized infectives.

The balancing constants justifying the level of importance of one type of intervention over the other are chosen as $W_1=1,\ W_2=0.7, \ W_3=0.3$ and $W_4=0.5$. The cost coefficients corresponding to the individual vaccination and intensive treatment are considered as $B_1=250,\ B_2=250,\ B_3=250$ and $B_4=1000$, indicating that the treatment cost for COVID-19 is very high as compared to the cost of vaccination. According to the parameters value given in Table 3 and the above mentioned initial conditions, the graph of control profiles is shown in Fig. 8. Using the control mechanism the solution trajectories that are formed corresponding to the model system (2) with and without optimal controls, can be visualized in Fig. 9. It can be seen from Fig. 9, the proposed optimal combination of both vaccination and treatment of COVID-19 infectives is proved to be very effective in reducing the infection prevalence and the financial burden on the government and individuals.

From the representation of control profiles (see Fig. 8), it can be observed that to reduce the infection prevalence, vaccination of individuals exposed to COVID-19 is required with full intensity, whereas the vaccination of undetected infectives with COVID-19 must be provided with its full intensity by the first 27 days and may decrease gradually to control the cost of vaccination. On the other hand, as our aim is to reduce the vaccination cost along with the infectives, it is optimal to provide vaccination to the susceptibles only for the initial days with its full intensity as they must have natural immunity in their body to fight against the coronavirus. Then the intensity should be decreased gradually to keep the cost as low as possible. However, since individuals under the treatment in hospitals may also infect other health care employees, therefore, only a few number of severely infected individuals who require intensive care needs to be provided the health care facilities in the hospitals and others must be stayed under the self-isolation or isolation centers established by the health care authorities. The treatment profile indicates that the treatment is required with its full intensity for the initial $4{-}5$ days, after which the treatment intensity should be decreased slowly till the end of 30 days. However, the treatment will not be provided with its full intensity till the end of study period as the COVID-19 treatment cost (including ICU and ventilator cost) is relatively expensive to the vaccination cost and health care workers are also prone to acquire infection due to overcrowding of infectives in hospitals. Thus, the optimal treatment strategy is to provide vaccination with higher intensity specifically to the individuals suffering from other immune based comorbidities and exposed individuals till the end of study period and reduce the treatment intensity gradually with respect to time by motivating infectives towards the self-isolation.

**Figure 10:**
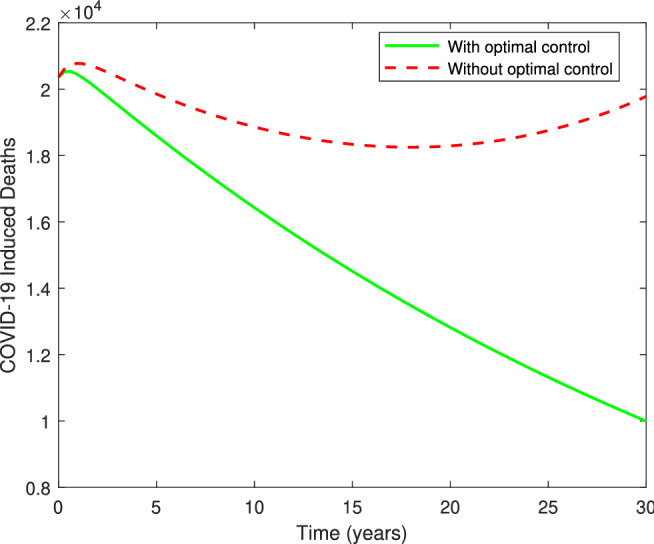
Graph illustrating COVID-19 induced death cases with and without optimal controls.

Figure 9, illustrates the behavior of solution trajectories with and without optimal controls (for the fixed value of $u_1=0.00016, u_2=0.00005, u_3=0.00005$ and $u_4=0.3$). From Fig. 9(a), it can be observed that susceptibles decrease significantly when the vaccination is applied at an optimal level and move to the class of vaccinated individuals which increases the population level in the vaccination class as can be seen in Fig. 9(c). From Fig. 9(b), we can observe that the number of exposed individuals increases at a higher rate when vaccination is not provided with its full intensity. Figure 9(d) shows a remarkable decrease in the class of undetected individuals when vaccination is applied at an optimal level. Thus, increasing the vaccination rate is very important to control the spread of coronavirus(COVID-19) infection from undetected infectives, which is a main cause behind community transmission. Also, Fig. 9(e) shows that the number of infectives in isolation centers decreases significantly when optimal control strategies are applied as compared to the case when vaccination and treatment are not applied at an optimal level. This may happen due to the fact that isolated infectives are getting hospitalized at an optimal level in comparison to the case when optimal strategies are not applied. Whereas, the number of individuals taking intensive care in hospitals show a remarkable improvement for initial few days when the treatment is given at an optimal level (Fig. 9f). The hospitalized infectives start decreasing by the end of the study period as improvement in the vaccination rate decreases the number of infectives, and hence the number of hospitalized individuals. Figure 10 displays the dynamics of COVID induced deaths in both the cases, that is, with and without optimal control. It can be clearly observed that the number of COVID induced deaths is increasing till the end of 30 days when optimal controls are not applied. However, the number of COVID induced deaths is decreasing significantly when all the controls are applied as compared to the case when no optimal control is applied.

Thus, it can be concluded that to attain the maximum reduction in the infection prevalence, efforts in the direction of increasing the intensity of vaccination and treatment measures such as sufficient number of beds in hospitals, ventilator facilities and proper care must be done.

## Discussion and conclusion

The novel coronavirus(COVID-19) has become a destructive cause for both human health and economy across the world. It has already affected over 215 countries all over the world majorly to USA, Brazil and India. India has haphazardly faced the second wave of COVID-19 disease causing the deaths of more than 421 thousands infected people. As reported by the World Health Organization, no complete cure has been introduced yet against the COVID-19 infection. Also, many non-pharmaceutical precautionary measures such as wearing masks, body sanitization, social distancing and isolation of infectives are taken into consideration but are not proved to be very effective in reducing the transmission of COVID-19 infection. Hence, more than 12 million individuals have been infected from COVID-19 disease. To reduce the infection prevalence, combined efforts towards the vaccination of the population from COVID-19 and the treatment of infectives are required.

This paper is focused on a deterministic COVID-19 model by incorporating the vaccination for exposed, infected individuals and the treatment for COVID-19 infectives. Onto the model, the optimal control strategies are applied by introducing the time dependent parameters to optimize the number of infectives while keeping the cost of vaccination and treatment as low as possible. In the model, the vaccination is applied on three classes of population, that is, susceptibles, exposed and undetected infectives to eradicate the disease from the population. To analyze the dynamics of this model, the basic reproduction number ${\mathcal {R}}_0$ has been computed. For the threshold quantity ${\mathcal {R}}_0$, the effect of parameters on ${\mathcal {R}}_0$ is determined on the basis of sensitivity index. From the sensitivity index, it is observed that the transmission rate and the efficacy of vaccination are the most influencing parameters in controlling the spread of the disease. The disease-free equilibrium point and the endemic equilibrium point has been computed to determine the state in which the disease can be eradicated or becomes endemic in the population. Corresponding to the constant values of the parameters, the model has been numerically simulated to justify the behaviour of the solution trajectories in the neighborhood of the equilibrium points and the role of vaccination in controlling the spread of COVID-19 infection. Numerical simulations indicate that when the reproduction number is greater than unity, a unique endemic equilibrium point exists which is locally asymptotically stable. For ${\mathcal {R}}_0>1$, the unstable disease free equilibrium point exists. This justifies, if the threshold quantity crosses unity then the disease will spread drastically into the population. On the other hand, if ${\mathcal {R}}_0<1$ then two different endemic equilibrium points exist out of which one is locally asymptotically stable. Simultaneously, a locally asymptotically stable disease-free equilibrium point comes into existence. For the threshold quantity less than unity, the existence of COVID-19 endemic equilibrium points justify that it is not sufficient enough to reduce the reproduction number below one in order to eradicate the disease from the population, but the vaccination also plays a crucial role in reducing the spread of COVID-19 infection.

It has also been observed that after receiving the vaccination against COVID-19, vaccinated individuals still need to follow all the non-medicinal precautionary measures mentioned by the health care authorities such as proper hygiene, mask wearing, social distancing, breathing etiquette and avoid the intake of alcohol to increase the efficacy of vaccination, and hence decreasing the level of infection. The impact of optimal control interventions has also been observed in the numerical simulations to control the spread of COVID-19 disease. It has been observed that individuals exposed to COVID-19 and undetected infectives require the vaccination with its full intensity while the vaccination rate can be reduced for the susceptibles in order to reduce the financial burden on government and individuals. To reduce the number of infectives and increase the number of recovered individuals, the detected infectives must be encouraged to remain under isolation and may not transfer to the hospitals for treatment until they require intensive care. Accordingly, the risk of infection for the health care employees and the cost of intensive care treatment will remain under the control. The combination of all the optimal control strategies together provides a remarkable decrease in the number of exposed and infected individuals along with the COVID induced deaths with an increment in the number of vaccinated individuals.

Thus, it can be concluded that the transmission of COVID-19 disease can be controlled while minimizing the cost of vaccination and treatment provided the vaccination rate increases significantly and all the vaccinated and unvaccinated individuals people must follow all the non-pharmaceutical precautionary measures as mentioned by the health care authorities. The success of optimal control strategies mainly rely on the proper implementation of government policies by infectives and proper management by the health care authorities.
